# Silicon‐Rich Amorphous SiC_*x*_ for the Lithium‐Ion Batteries: How Does Strong Carbon Doping Affect the Lithiation Behavior and Electrochemical Performance?

**DOI:** 10.1002/cssc.202502723

**Published:** 2026-04-05

**Authors:** Moritz Loewenich, Hartmut Wiggers

**Affiliations:** ^1^ EMPI Institute for Energy and Materials Processes – Reactive Fluids University of Duisburg‐Essen Duisburg Germany; ^2^ CENIDE Center for Nanointegration Duisburg‐Essen University of Duisburg‐Essen Duisburg Germany

**Keywords:** amorphous, battery, carbide, silicon, stability

## Abstract

Amorphous, substoichiometric silicon carbide (a‐SiC_*x*_) is explored as an anode material for lithium‐ion batteries. Silicon‐rich a‐SiC_*x*_ nanopowders with different carbon concentrations yet otherwise comparable properties are produced and processed into electrodes. By electrochemical testing, performance data for the different materials are obtained, and in a second step, correlated with further information from detailed analysis of the lithiation and delithiation behavior as well as electrode morphology evolution dependent on the carbon concentration. An increased lithium loss in the first cycle can be linked to the carbon content in the sample, suggesting a matrix‐phase‐like behavior. In the first cycles, roughly 1 atom of lithium is lost per atom of carbon in the sample, and additionally, each carbon atom inertises up to 2 Si atoms. Cycling stability, on the other hand, is highest for 25.9 at.% C, with no capacity fade after 200 cycles and excellent rate capability. With a lower anode potential and reduced voltage hysteresis, especially during fast‐charging, the carbon‐rich samples show further benefits. Data from fast‐charging and impedance analysis indicate that the solid electrolyte interphase (SEI) remains stable over long‐term cycling for the carbon‐rich materials. Postmortem analysis confirms the assumption that the material is operating around a rather high lithiation state.

## Graphical abstract

In this article, amorphous substoichiometric silicon carbide (a‐SiCx), which is produced from a scalable gas‐phase process, is investigated on the influence of the carbon concentration on the electrochemical performance. Thorough testing in half‐cells combined with material analysis and postmortem studies reveals a promising material with virtually no capacity fade over cycling, reduced voltage hysteresis, and improved rate capability.

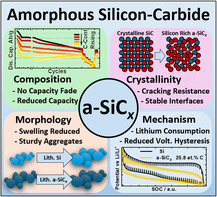

## 1 Introduction

Lithium‐ion batteries (LIB) are a central asset in the electrification of our societies, and optimization of their active materials is essential for enhancing their performance. On the anode side, graphite is the common active material and has a specific delithiation capacity of around 372 mAh/g when lithiated to LiC_6_ [1]. Limited by this low capacity, no further big improvements are to be expected in the use of graphite‐based anodes, hence, new materials are considered. One of them is silicon, first proposed in the 1990s [2, 3], but the widespread commercial use is just gaining traction in the last years [4]. Silicon is particularly promising since it has an enormous delithiation capacity of up to 4000 mAh/g when lithiated to Li_4.4_Si [5], offering up to 20% higher capacities of LIBs on cell level [6]. Since the cathode materials generally show a lower capacity, specific anode capacities of up to 1200–1500 mAh/g strongly improve the storage capacity on cell level, but higher values bring minor improvements [7]. However, silicon is plagued by various issues correlated with the high capacity, mainly caused by a volume expansion of up to 300% during lithiation [8], which limits the cycle life [9].

In lithiation, silicon particles can fracture under the enormous mechanical stress induced by the volume expansion. The resulting pulverization issue is well‐documented and can be mitigated by reducing the particle size to the nanoscale. Reducing crystalline Si particle diameter below ≈150 nm prevents fracture during cyclic lithiation [10] and simultaneously diminishes voltage hysteresis and therefore enhances the overall energy efficiency [11]. However, around 150 nm diameter, the specific surface area of the powder is in the range of 20 m^2^/g, which leads to a significant increase in parasitic surface reactions and thus reduces the coulombic efficiency (CE) in the first cycles. Furthermore, the handling of the powder as well as slurry preparation with high solid content is becoming increasingly difficult, next to rising safety concerns with decreasing particle sizes below 100 nm [12, 13, 14].

The limitation regarding particle size can be overcome by switching from crystalline to amorphous silicon (a‐Si), where the particle size limit regarding fracture increases to about 500 nm [7, 15], while the specific surface area is reduced by around 70% compared to 150 nm particles. The main reason for the higher fracture resistance of amorphous silicon is its isotropic lithiation, whereas the rate of the initial lithiation in crystalline particles is dependent on the crystal orientation, causing anisotropic strain [16, 17]. Moreover, crystalline particles tend to be lithiated with a sharp local gradient in lithium concentration from the shell to the core, due to the formation of specific Li_*x*_Si_*y*_ phases. Amorphous materials can be lithiated more homogeneously in a one‐phase reaction [14, 18]. Furthermore, a‐Si has a lower hardness and Young's modulus, enabling it to deform plastically and thus absorb volume changes without cracking [19]. Amorphous silicon is additionally slightly less dense than crystalline (about 3%) [20], yet this is not expected to have a big influence on fracture resistance.

In addition to offering greater flexibility regarding maximum particle size, the amorphous structure offers several benefits. Because a‐Si is lithiated as a single, continuous phase and does not undergo the crystalline to amorphous transformation, it also avoids the steep Li‐concentration gradients and stress concentrations that develop at moving phase boundaries in crystalline Si. This more uniform lithiation, together with inherently faster Li^+^ diffusion [21], translates into significantly improved rate performance. While this improved rate performance is typically not relevant in the formation cycle, recent findings indicate that parts of the crystalline structure may be retained over many cycles, especially for oversized anodes [16].

To improve the stability of Si‐based anodes, other solutions are being offered in literature, for example, coating of the silicon mostly with carbon [22, 23], advanced composites [23], different Si‐morphologies [24, 25], electrolytes [26, 27], electrode structuring [10, 11, 28], prelithiation [29, 30], advanced binders [15, 31], or adapted cycling [32, 33]. However, beyond tuning particle size, the material properties of the silicon present an avenue for exploration, which is seldomly methodically investigated. Our work addresses this gap in knowledge.

Another issue related to mechanical stress due to phase changes is the nucleation of crystalline Li_15_Si_4_ at high lithiation, especially observed for crystalline silicon. This phase has been reported to be detrimental due to parasitic currents [34] and an increased reactivity with the electrolyte [19]. It has been shown that the formation of this phase can be prevented by heavy doping/alloying of heteroatoms into the Si‐network such as carbon [21, 35], nitrogen [36, 37], oxygen [38, 39], germanium [40], aluminum [41], boron [42], etc., or their combinations. Periodic interferences with the lattice disrupt long‐range order and thus prevent crystallization, avoiding the formation of Li_15_Si_4_ upon cycling, thereby stabilizing the structure and extending cycle life.

Although the addition of dopants or alloying elements decreases the specific capacity of silicon, it has been shown that its long‐term cycling stability can be greatly increased. This opens up the parameter space of the heteroatom concentration for optimization and fundamental understanding of its effect. The increased cycling stability is often attributed to conversion‐type reactions, where stable compounds are formed, including silicon, the heteroatom, and lithium, limiting further solid electrolyte interphase (SEI) growth [43], accompanied by a reduced volume expansion [44]. While these benefits can also be reached by only partial lithiation of the silicon, employing a doped material which intrinsically has these properties will be a more reliable option and does not require precise potential controls at all times. In fact, Obrovac et al. showed that the dilution of silicon by heteroatoms can even increase the specific energy compared to a partial lithiation achieving the same volume expansion, since the lower anode potential on average yields a higher average cell voltage [44].

Of the above‐mentioned doping/alloying elements, only very few are suitable for large‐scale industrial applications for various reasons: Despite its promising properties, that is, increasing the lattice constant enabling faster lithium diffusion [40], germanium is unsuitable for cost reasons. Al and B can only be incorporated in small percentages, and even the frequently used SiO_*x*_ has limitations due to challenges regarding electronic and ionic conductivity, which prevents its use in double‐digit percentages in the anode. In contrast, carbon is ideal as a heteroatom because it is isovalent and can therefore be more easily incorporated in the lattice. Moreover, it is easily available as a precursor, abundant and can also reduce the material density, which in turn has a favorable effect on gravimetric energy density. In this study, we therefore conducted a systematic investigation into the production of substoichiometric SiC_*x*_ and its electrochemical properties. The nanoparticles (NP) studied are produced in a gas phase process, specifically a hot wall reactor, from monosilane and ethylene where the particle composition is well controlled, while other particle properties can be mostly kept constant. While the production of this material and scaling up to the kg/h range has already been extensively researched and forms the basis for the materials produced [21, 35], this study focuses on the systematic investigation of the electrochemical properties of a‐SiC_*x*_ as a function of chemical composition, whereby the particle size was selected to meet the above criteria for preventing mechanical decomposition. A wide range of materials is tested in half‐cells against Li‐metal to study the differences in capacity and CE. Anode potentials are tracked regarding position of lithiation and delithiation peaks and their stability during cycling, giving information on material‐dependent voltage hysteresis and buildup of resistances. By linking results of postmortem electrode analysis and electrochemical characterization, including electrochemical impedance spectroscopy (EIS) and galvanostatic intermittent titration (GITT), the lithiation and de‐lithiation behavior of the a‐SiC_*x*_ materials depending on the carbon content can be thoroughly described.

## 2 Material and Methods

### 2.1 a‐SiC_*x*_ Nanopowder Samples

The particles investigated in this study are produced in a custom‐built pilot‐scale hot‐wall reactor, described in detail in previous publications [35, 45]. The main reaction is the pyrolysis of monosilane to hydrogen and silicon, whereby the decomposition of ethylene is catalyzed by the decomposition products of monosilane. Batches of 200 g were produced in 30 min experiments at 790°C furnace temperature. This temperature is chosen, since at lower temperatures the monosilane and ethylene consumption may be reduced, while undesirable crystalline areas could form at higher temperatures. A monosilane concentration of 20% was used, and the residence time in the hot zone was fixed at 5 s, assuming a plug flow approximation. Comprehensive material characterization is reported in [21]. For this study, samples with a carbon content ranging from 0 to 37 at.% are produced and analyzed by varying the ethylene to monosilane ratio from 0 to 0.5.

### 2.2 Powder Characterization

To determine the particle size and morphology, transmission electron microscopy (TEM) was applied with a Cs corrected TEM (JEOL, JEM‐2200FS) operated at 200 kV. The samples were prepared by dispersing the NPs in ethanol and drop casting the suspension on a TEM grid. For statistical reliability, at least 300 particles were analyzed regarding their primary particle diameter to obtain a particle size distribution (PSD). The specific surface area was measured via the Brunauer–Emmet–Teller method (BET) in a Quantachrome NOVA 2200. Prior to BET analysis, the powders were dried in vacuum at 120°C for 16 h to remove physisorbed water.

To investigate the crystal structure, the powders were analyzed using X‐ray diffraction (XRD, PANanlytical, X’Pert) with Cu K_α_ radiation. Bulk hydrogen, oxygen and carbon were quantified by elemental analysis (ELTRA, ONH‐p and ELTRA, CS‐I, respectively) via hot gas extraction method.

### 2.3 Electrode Preparation

To assess the performance of the powders in a LIB, half cells versus lithium were built. Slurries were prepared consisting of the silicon‐based active material, polyacrylic acid (PAA, 25 wt.% in water, M_w_ = 240,000 g/mol, Alfa‐Aesar) as a binder, and conductive carbon (Super C65, Imerys) in a ratio of 75/15/10 by weight, respectively. The total solid content of the slurries was typically set around 40 wt% in water. Slurries made from total 2 g of solids were homogenized with three 4 mm Zirconia balls in a centrifugal mixer (Are‐250, Thinky). The slurries were cast on a copper foil with a wet film thickness around 50 µm. After drying at 60°C, 12 mm discs with a mass loading between 0.8 and 1 mg/cm^2^ were selected for testing. Three Swagelok type T‐cells were built for each material using 120 µl of electrolyte (1 M LiPF_6_ in a 1:1 ethylene carbonate (EC), dimethyl carbonate (DMC) mixture with 5 wt.% fluoroethylene carbonate (FEC) as an additive). Lithium foil was used as counter and reference electrode and installed together with glass fiber separators (GF/D, Whatman).

### 2.4 Electrochemical Analysis

A constant‐current constant‐voltage protocol was applied for cycling at 25°C between 0.01 and 1.2 V versus Li/Li^+^, with one 0.05 C and one 0.1 C formation cycle and further cycling at 0.5 C for charge and discharge using a Maccor 4000 battery tester.

Further electrochemical analysis was performed in the same cell setup. EIS was recorded after every cycle at lithiated (not shown) and de‐lithiated state, over a frequency range of 100 kHz to 0.05 Hz with 10 mV signal amplitude after 30 min waiting time, using a high‐precision potentiostat (VMP‐3, Biologic). The spectra were analyzed using the RelaxIS software (rhd‐Instruments) and proprietary software VisFit. Total test procedure consists of 5 cycles at C/10, and 5 cycles at C/2, followed by a GITT protocol with 15 min C/10 pulses and 30 min relaxation (1 cycle). Afterward a cyclovoltammetry protocol with scan rates between 10 and 500 µV/s (6 cycles, 1 per scan rate), and 50 cycles at C/2 is conducted to assess electrode degradation. From GITT, the diffusion coefficient is estimated using Equation (1), with the diffusion coefficient *D*, the molar volume *V*_m_, the Faraday constant *F*, the electrode area *A*, the applied current *I*, the voltage at the end of rest period *E*_4_ and at the beginning of the current pulse *E*_0_, as schematically shown in Figure SI 6 [46]. For the CV evaluation, Equation (2) is used, with *d* *j*/*d* *ν* ^0.5^ as the slope of the peak currents *j* plotted over the square root of the scan rate *ν*, *n* as the charge concentration in Li_*x*_Si (adapted for lower capacity of a‐SiC_*x*_), *A* as the electrode area and *C*_Li_ as the concentration of lithium in the electrode [47]. Based on the diffusion coefficient, diffusion times can be estimated by Equation (3) with the characteristic length *L*.

(1) $$\text{GITT}:D_{{\mathrm{Li}}^{+}}=4{\left(\frac{V_{m}}{FA}\right)}^{2}\frac{I}{\pi }{\left(\frac{E_{4}-E_{0}}{\frac{dE}{dt^{0.5}}}\right)}^{2}$$

(2) $$\mathrm{CV}:D_{{\mathrm{Li}}^{+}}={\left(\frac{dj/d\sqrt{\upsilon }}{2.69\cdot 10^{5}n^{3/2}AC_{\mathrm{Li}}}\right)}^{2}$$

(3) $$\tau_{\text{diff}}=\frac{2L^{2}}{D}$$

### 2.5 Electrode Characterization

The thickness of the coated electrode layers was measured with a micrometer gauge (±1 µm accuracy), and their electrical resistance was measured by mounting the electrode between two stainless steel cylinders under an uniaxial pressure of about 0.6 MPa. The resistance was determined by applying a current of 1 mA and measuring the steady‐state voltage drop. For each material produced, 3 different electrodes were measured with each tested 5 times, and the results were normalized to the mass‐loading of the respective electrodes.

The morphological analysis of pristine and cycled electrodes was carried out by scanning electron microscopy (SEM, CLARA, TESCAN) at 5 kV to investigate surface and cross sections of pristine and cycled electrodes. In a first step, the electrodes were weighed before and after cycling and left lithiated/delithated after 1 or 3 cycles. For each point, duplicates were produced, resulting in 8 cycled electrodes per material investigated. After cycling, the electrodes were rinsed twice in EMC for 20 s each and then dried. Flat cross‐sections were prepared by argon milling (IB195030 CP, JEOL) to determine the porosity and morphology of the cross‐section of cycled electrodes.

## 3 Results and Discussion

**FIGURE 1:**
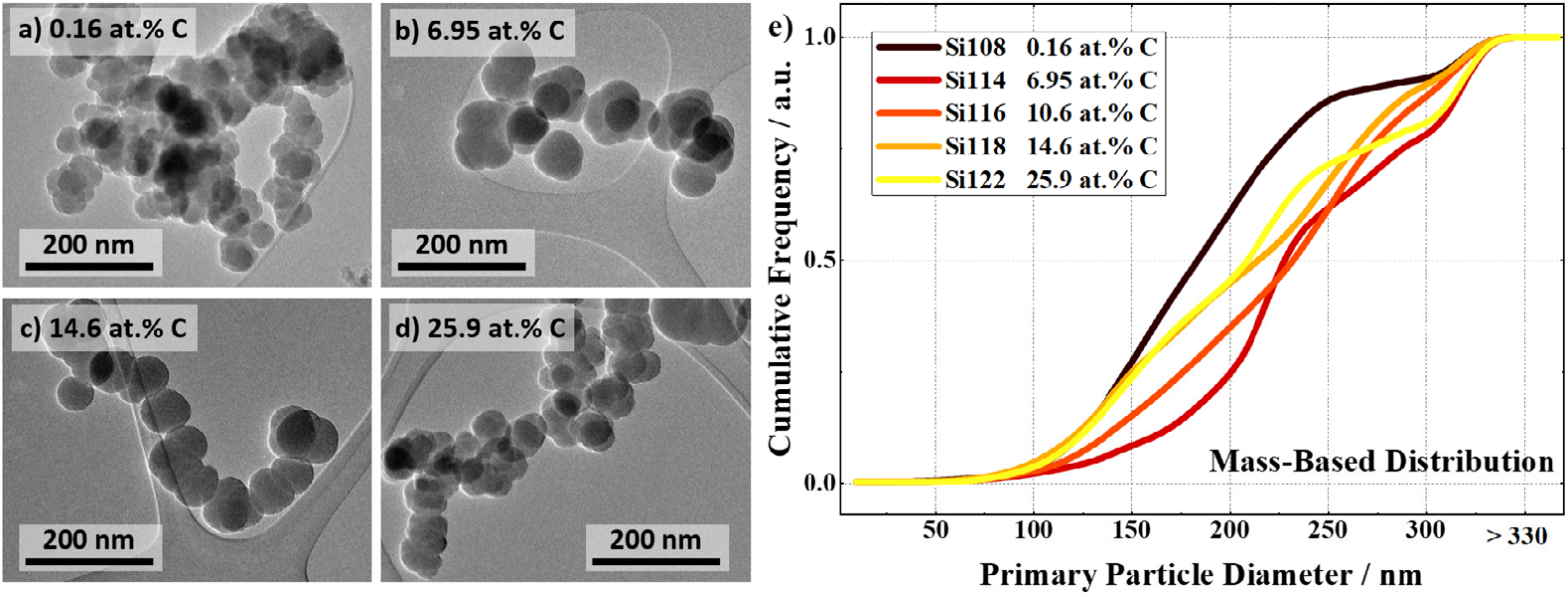
(a–d) TEM micrographs of 4 selected a‐SiC_*x*_ materials with various carbon contents, (e) corresponding evaluation of the mass‐weighed cumulative primary particle size distribution. Size evaluation is described in more detail elsewhere [21].

In order to systematically investigate the influence of carbon content on electrochemical performance and material properties, 18 different powders with a carbon content between 0 and 37.4 at.% C were produced in the hot‐wall reactor described above. The color of the pristine powders changes from nearly black for pure silicon to a saturated brown for materials with high carbon contents. With all process conditions fixed except for the C_2_H_4_:SiH_4_ ratio, this color change is expected to be a composition effect, not size or crystallinity. The results of powder diffraction, BET, and carbon analysis of all samples are summarized in Table SI 1. Given that the reactor temperature was identical during the synthesis of the different materials, it is not surprising that the BET surface area determined shows only minor variations. As already mentioned in the introduction, the successful incorporation of carbon can significantly reduce the crystallinity of the manufactured materials. Thus, the amorphous fraction of the powders quickly rises to >99% for materials with > 4 at.% C, as determined by XRD, results shown in Figure SI 1. Morphologically, the powders are alike, as observed in TEM in Figure 1a–d. While the primary particles are spherical, the powder consists of strongly aggregated structures. Only for the pure silicon material, a bit of texture is visible inside the particles, confirming a partly crystalline structure. The cumulative mass‐weighed PSDs obtained from the evaluation of histograms of TEM images are shown in Figure 1d. Except for pure silicon (d50 = 184 nm), the count median diameter of the primary particles in all samples is between 210 and 230 nm. This lets us ascribe the effects we observe regarding the electrochemical performance mainly to the carbon content. It has been found that with rising carbon content, the degree of disorder in the system is increased, while carbon is introduced into the silicon lattice together with hydrogen. Importantly, the carbon in these samples is not present as a coating layer, but distributed on the atomic level in the silicon material. More details on the material properties in dependance on the carbon content are presented in literature [21].

### 3.1 Electrochemical Screening

To assess the performance in the battery and understand the influence of carbon incorporation, in a first step, the analyzed powders are produced into electrodes. Compared to pure silicon powders, the apparent hydrophobicity is slightly increased with a higher carbon content, also the amount of water needed to obtain a slurry with an appropriate viscosity. Typical solid contents in the slurry are 40–50 wt.%. Polyacrylic acid is a common binder for silicon‐based anodes with oxidized surfaces. Since the oxygen content of the materials presented here is comparable to that of pure silicon, a good adhesion is expected and found for all electrodes. Prior to the electrochemical measurements, all prepared electrodes were examined for mechanical stability and electrical conductivity to determine the extent to which the carbon content has an effect here. The results are presented as boxplots in Figure SI 2, with 15 measurements per sample. The trend toward slightly improved conductivity with higher carbon content should not be overinterpreted because the changes are within the margin of error. We assume that the conductivity is essentially determined by the conductivity additive; in any case, insufficient conductivity for materials with high carbon content can be ruled out. The adhesion of the layers is deemed sufficient for all materials by a simple scratching test.

**FIGURE 2:**
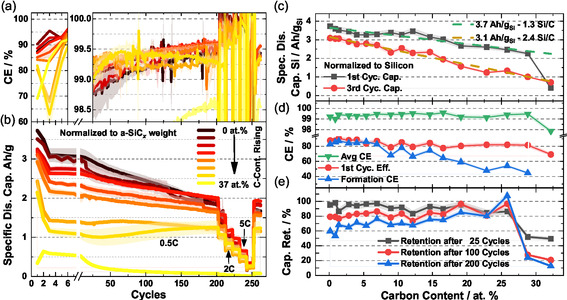
Electrochemical test data from half cells against Li metal with anode materials of different carbon composition. (a) Coulombic efficiency over cycle number. (b) Specific discharge capacity over cycle number. In cycles 200–260 a rate test up to 10 C is performed. Right side: Evaluation of (a,b) plotted over the carbon content of the anode active material. (c) Specific discharge capacity of first and third cycles normalized to silicon content. Two lines are fitted to these curves to describe the reduced capacity with rising carbon content. (d) First‐cycle coulombic efficiency, average coulombic efficiency (cycle 4–200) and the average coulombic efficiency of the first 3 cycles. (e) discharge capacity retention after 25, 100, and 200 cycles with respect to the third cycle capacity.

For all materials, half cells versus Li/Li^+^ are built and tested over 262 cycles in total. The resulting coulombic efficiencies and specific discharge capacities are plotted in Figure 2a,b, with the corresponding evaluation regarding the carbon content of the anode active material in c–e.

While for pure silicon the capacity starts high at 3.7 and 3.1 Ah/g in the first and third cycles, it decays rather fast to 1.5 Ah/g after 200 cycles. With rising carbon content, the initial capacity is decreased, while the decay becomes slower. For some materials around 20–25 at.% C, the capacity retention is completely stabilized over 200 cycles, whereas the capacity is reduced sharply for carbon contents higher than 27 at.% C. The rate test from cycle 200–260 shows the rate performance at 1C, 2C, 3C, 5C, and 10C. At 2C, most materials still show a capacity around 1 Ah/g, while at 5C, for most materials, 0.5 Ah/g are left. After the rate test, the capacity from before at 0.5C is retained.

Figure 2c shows more detailed, how the initial discharge capacity and the discharge capacity in the third cycle at 0.5 C are developing with respect to the carbon content. The shown capacities are normalized to the silicon content. This means that if the entire amount of silicon in the material would always be fully lithiated, one would expect a horizontal line. However, the first cycle of pure silicon shows the theoretically expected 3.7 Ah/g_Si_, while a material with 26 at.% C shows around 2.2 Ah/g_Si_. In between, a semi‐linear behavior is observed. The corresponding slope suggests that each incorporated carbon atom leads to an average of 1.3 silicon atoms that are no longer available for lithiation. The corresponding trendline is shown in the graph in green. For the third cycle at 0.5 C, the delithiation capacity is reduced from 3.1 Ah/g_Si_ for pure silicon, to 1 Ah/g_Si_ for a material with 26 at.% C. Here, a similar analysis shows that each introduced carbon atom leads to, on average, 2.4 silicon atoms not taking part in the reaction. While the different slopes cannot be explained in detail here, the results are interpreted as follows: i) the carbon is not considered as a species that can be lithiated, since it does not form graphite‐like layered structures; ii) the carbon atoms are not just present as an inert constituent not taking part in the lithiation, but the silicon‐carbon environment seems to be less susceptible for lithiation, withstanding lithiation up to 0.01 V against Li/Li^+^. Following this argumentation, then each carbon atom could inertise up to 4 silicon atoms. Yet in a previous work, it was found that in this production method carbon is typically inserted together with hydrogen, reducing the available bonds of carbon with silicon. We explicitly point out that standardization to the Si content was only performed for this representation; in all other graphs, the total amount of a‐SiC_*x*_ (including the carbon) is considered. In literature, sometimes even for pure SiC (50 at.% C) capacities between 300 and 800 mAh/g are reported, which would be higher than for the materials shown here, if the line in Figure 2c would be extrapolated [48, 49]. As an explanation, it is assumed that mostly no stoichiometric SiC phases are formed in the present material, because the carbon atoms are not present in a concentration high enough, so the underlying lithiation mechanisms are fundamentally different to SiC lithiation.

Looking at the coulombic efficiencies in Figure 2d, it is visible that the FCE and especially the cumulative CE of the formation (first 3 cycles, blue graph) is strongly materials dependent. While the cumulative CE of the formation is at 80% for materials up to 7.5 at.% C, it is decreased down to 50% for materials with 20–26 at.% C, making prelithiation of these materials absolutely essential for a potential commercial application. Since almost no differences in particle morphology and specific surface area of the materials were observed, we ascribe the CE drop to the volume properties of the materials. This is further supported by the fact that there is still a decreased CE in the second cycle. Possible explanations would be irreversible reactions inside the material, involving carbon and lithium, that is, some Li_*x*_SiC species [49]. Looking at the atomic concentrations, it can be said that taking the 80% formation Coulombic efficiency for pure silicon as a baseline loss, for each atomic percent carbon added, the CE of formation is reduced by one percent, as shown in Figure SI 3. This can be translated to the loss of 1–2 lithium atoms per carbon atom introduced. While no measurements to confirm Li_*x*_SiC species can be provided, in literature, the formation of a Li_4_C species is reported as a result of a conversion reaction from SiC [50]. The average CE over long‐term cycling, on the other hand, is slightly increased from for 99.3% to 99.5% for materials with a higher content of carbon, indicating a high interfacial stability, and no further ongoing irreversible bulk reactions.

The capacity retention compared to the third cycle, presented in Figure 2e, shows two major trends. After 25 cycles, most of the materials exhibit a good capacity retention around 90%, only the materials with higher carbon contents are a bit lower around 85%. Yet after 200 cycles, the trend is reversed. While materials with little carbon show retentions around 70%, high carbon materials up to 26 at.% C show a retention even over 100%. This behavior of slowly rising capacity is observed also in literature for similar materials and is typically correlated with slow restructuring of the material, presenting more available silicon over time [50, 51]. One possible cause here is the segregation of carbon and silicon.

Since this high retention, associated with a long lifetime, is a fascinating material property, the reason for this high cycling stability shall be investigated in more detail in the following. It is noteworthy to mention that these results were achieved in a high silicon loading electrode without graphite, with full lithiation, and with only standard processing and battery components.

### 3.2 Electrode Potential Analysis

**FIGURE 3:**
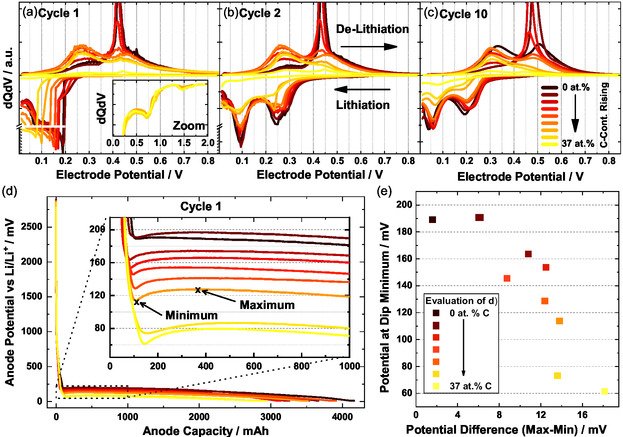
dQdV plots of a‐SiC_*x*_ materials with different carbon contents; The black graph shows pure silicon, red medium carbon concentrations and yellow high carbon concentrations up to 37 at.%. (a) For the first cycle (C/20) with an inset zoomed on the high potential region typically associated with electrolyte decomposition [52], (b) for the second cycle (C/10), and (c) for the 10^th^ cycle (C/2). (d) Potential versus capacity plot for different materials (first cycle) with a focus on the local minima occurring around 100–150 mV versus Li/Li^+^. (e) Evaluation of the local minima regarding position versus potential difference between “Minimum” and “Maximum” as shown in (d). All plots show average profiles of 2–3 Cells.

To gain further understanding of the electrochemical processes occurring in the anode, differential capacity plots are created. Changes of lithiation or delithiation mechanisms may be visualized here by potential differences. Plots for all materials for Cycle 1, 2, and 10 are depicted in Figure 3, together with an anode potential – capacity plot and its evaluation.

Looking at cycle 1, we see the lithiation of the pure silicon starting at an anode potential of 195 mV (black graph Figure 3a). For materials with medium carbon content, the potential shifts to 150 mV, and for 37 at.% C, it only reaches 85 mV versus Li/Li^+^. A voltage drop due to the electrical resistance (IR‐drop) on electrode level is not to expected in this magnitude because of low mass loading and low current densities in the first cycle (≈0.1 mA/cm^2^). Furthermore, no increase in electrical resistance was found for pristine electrodes with carbon‐rich materials (Figure SI 2). Thus, we attribute this shift to an activation overpotential due to the electrochemical reaction or to a concentration overpotential due to diffusion‐limited processes. The decrease in the lithiation potential by 110 mV could have various reasons. Reasons could include the formation of a strong SEI, leading to an overpotential on particle level, or a bulk property of the carbon‐rich material due to a less favorable lithium insertion at higher carbon concentrations. The latter would fit with the assumption of lithium insertion being less favorable in carbon‐containing environments, as stated above. Regarding SEI formation, the inset in Figure 3a shows a zoom of the typical area of electrolyte decomposition around 0.75 V. There are no significant differences here, so an increase in potential drop due to increased SEI formation can be ruled out. The second option is explored in more detail, analyzing a special feature in the voltage‐capacity plot in Figure 3d, a dip in the voltage (labeled ‘Minimum’ in the inset), followed by a rise afterwards (labeled ‘Maximum’). This feature is not visible in the differential capacity plots due to data processing for averaging of multiple profiles. Typically, such a behavior correlates with an activation of the material, that is, the conductivity or Li^+^ diffusion of the material is increased by initial lithiation, or the core material is made available for lithiation. Since the core of the particles is richer in silicon compared to the shell [53], the latter explanation fits well with the observed phenomena. Figure 3e shows a deeper analysis of the activation phenomenon. The potential at which lithiation starts (*y*‐axis) is correlated with the carbon content and the potential difference between ‘minimum’ and ‘maximum’. It is found that for higher carbon contents and potentials at which the lithiation starts, the potential difference is increasing from 2 to over 16 mV. Since conductivity limitations are unlikely in the first cycle, the most probable explanation here is an activation mechanism of the more silicon‐rich core.

Looking at the de‐lithiation in the first cycle, Figure 3a shows a sharp peak around 425 mV, which is ascribed to the delithiation of the crystalline c‐Li_15_Si_4_ phase [19], the formation of which is detrimental for battery performance and earlier works already showed that the formation of this phase can be prevented effectively by the incorporation of carbon [53]. With our experiments, we can narrow down the carbon concentration needed to ≈10 at.%, since the material with 10.6 at.% already reliably prevents the formation of that phase. We attribute this to the amorphousness of the material caused by a strong lattice distortion due to the high amount of carbon atoms. This also applies to subsequent cycles, as shown in Figure 3b,c. After the material re‐formation in the first cycle to completely amorphous, the peak positions in the second cycle are significantly more similar between the different materials, also for the further cycles, none of the voltage dips shown in Figure 3d are observed. This suggests that the particles stay activated, maybe by a part of the lithium staying in the particles, making them retain a higher degree of electronic/ionic conductivity. This also would combine with the observation of lower CE for materials with higher carbon content.

Furthermore, the stability of the lithiation peak positions will be analyzed by comparing the second cycle, presented in Figure 3b, with the 10^th^ cycle, presented in Figure 3c. For pure silicon, the peaks shift quite substantially from 95 and 250 mV to 45 and 195 mV, while carbon‐rich materials shift from 90 and 225 mV to 75 and 175 mV. Also, for the further cycles, pure silicon keeps shifting to lower electrode potentials, while carbon‐rich materials are lithiated at the same potentials up to 200 cycles (Figure SI 4). A similar trend can be observed for the de‐lithiation, just now with pure silicon drifting away to higher electrode potentials over cycling. The plots ordered by materials are available in Figure SI 4, until cycle 260.

The interface stability of carbon‐rich materials is further confirmed by the stability of the peak positions. A shift of lithiation peaks can be attributed to an overall rise in impedance, for example, by uncontrolled SEI growth for carbon‐poor materials, causing an IR‐drop, both on the electrode and particle level. An additional negative consequence of this IR‐drop is that the round‐trip efficiency of the battery is decreased when lithiation and delithiation peak split apart further, since charging is done at higher voltage, and discharging at a lowered one. This effect is also known as voltage hysteresis in the literature. While the described effects are based on the electrical resistance, also material properties can have an impact here. Recent publications link it to the plastic deformation of SEI during volume change, which causes internal stress. These stresses are linked to the lithiation potential of the silicon material [54]. For experiments on half‐cell level, this can be expressed as the average potential difference between lithiation and delithiation of the working electrode. A related quantity is the contribution of the constant current phase after lithiation to the total charge of that cycle, both plots are presented in Figure SI 5. While the average potential difference is around 300 mV for carbon‐rich materials, pure silicon shows values around 350 mV. This difference is sustained over cycling, yet in the rate test, it is growing even bigger for carbon‐poor materials. Here at 5C the average potential difference is only 525 mV for carbon‐rich materials, while for pure silicon and carbon‐poor materials it reaches 800 mV. These results further suggest that an impedance rise during cycling is prevented effectively for the carbon‐rich materials. A possible explanation could be a reduced volume expansion of the single particles due to the lowered capacity, which also prevents the frequent breakup and re‐formation of the SEI and reduces internal stress. Otherwise, the formation of a stable and conductive matrix phase would be another possible explanation at this point, next to the lithiation of a‐SiC_*x*_ being kinetically favored over Si.

**FIGURE 4:**
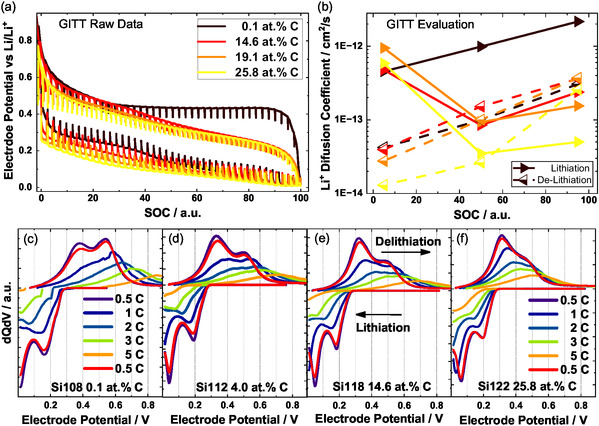
(a) GITT raw data for 4 different carbon concentrations, (b) evaluation of the Li^+^ diffusion coefficient dependent on SOC of data shown in (a). Differential capacity plots for material with (c) 0.1 at.% C, (d) 4 at.% C, (e) 14.6 at.% C and (f) 25.8 at.% C, measured at different C‐rates ranging from 0.5C to 5C; After the rate test, another cycle at 0.5C is shown in red for assessment of aging of the cell. Arrows indicate direction of lithiation and delithiation.

To understand if the changes of the material have not only have an impact on the kinetics, but also on the equilibrium potential, the quasi‐equilibrium potential curves in dependance of the state of charge are shown in Figure 4a, together with the evaluation of the diffusion coefficient (extracted from GITT as described in [46, 47, 55]) for delithiated, half‐lithiated and lithiated state shown in Figure 4b. For this analysis, one sequence consists of a current pulse followed by a rest period, as shown in Figure SI 6. For each sequence, the difference of the potential at the beginning and end of the sequence is divided by the slope of the potential versus the square root of time. The GITT curves show that both the charging curve and the equilibrium‐curve show a decreased voltage hysteresis in quasi‐equilibrium for carbon‐containing materials. Additionally, the state‐of‐charge dependent potentials of lithiation and de‐lithiation are generally lower for the carbon‐containing samples. A positive side effect is that lower anode potentials lead to an increased cell voltage, which is highly beneficial for the energy density of a battery cell. The obtained diffusion coefficients in Figure 4b are in the range of 10^−12^ – 10^−14^ cm^2^/s with generally higher values for low lithium contents during lithiation (around 10^−12^ cm^2^/s), and higher diffusion coefficients for high lithium contents during delithiation (around 10^−13^ cm^2^/s). It is noticeable that pure silicon has a significantly higher diffusion coefficient than carbon‐containing materials when charging. Based on this, average diffusion times through the particle can be estimated. For a 200 nm particle, these are in the range of 2 min for 5·10^−13^ cm^2^/s, clearly not diffusion limited, and 17 min for 5·10^−14^ cm^2^/s, still slightly below 30 min charge and discharge time. Based on the diffusion coefficients determined, we assume that there is no diffusion limitation for the materials for most working points examined here. This is in line with the previous results, since a diffusion limitation would suggest slower kinetics for the carbon‐rich samples, which was not found. The GITT results are further supported by cyclovoltammetry where we evaluated the peak current dependency on the scan‐rate to also determine diffusion coefficients [47]. Values between 2.2·10^−13^ cm^2^/s for pure silicon and 3.3·10^−12^ cm^2^/s for the material with 19.1 at.% C were found (Figure SI 7 & Figure SI 8). While here a faster diffusion, as well as an opposite trend was found compared to the GITT‐evaluation (since the diffusion here may not be the rate‐limiting step as discussed before)‐, these results may be less significant.

The rate test at the end of the cycling protocol (Figure 2b) can give an indication of the fast‐charging capability of the materials. Differential capacity plots for four materials are shown in Figure 4.

Looking at the fast‐charging test in Figure 4c–f, generally, before interpreting the data, it has to be noted that ‐ when comparing pure silicon to the carbon‐rich material ‐ the same C‐rates cannot directly be compared, since the silicon has a higher overall capacity, leading to a higher current at the same C‐rate. Assuming electrical or ionic conductivity can become the limiting factor, this causes higher IR‐drops, shifting peaks apart from each other. Furthermore, for pure silicon, due to capacity fading (60% retention after 200 cycles), nominal 3C charging is already effectively 5C charging. Taken together, the 2C plot in c) should be compared to the 5C plot in f). While the lithiation part for this comparison is not showing great differences during the delithiation, differences are visible. First, for pure silicon, the distinct delithiation peak of the Li_15_Si_4_ phase is not visible, which suggests an incomplete lithiation, or an increased influence of the SEI, or immobilized lithium in the particle core. Generally, for higher C‐rates, lithiation and delithiation peak drift apart from each other, which is a combination of increased IR‐drop in the anode layer and lithiation kinetics of the particle itself. If the volume lithium content cannot follow the surface lithiation due to slow lithium diffusion through the SEI or the bulk, the signal will shift in the dQdV plot, or the half‐cycle may be ended prematurely by the end condition. While for pure silicon the delithiation peak position is at 650 mV at 2C, for carbon‐rich material it only has increased to 550 mV at 5C. The materials in between show a rather linear shift from the extremes.

### 3.3 Postmortem Structure Analysis

**FIGURE 5:**
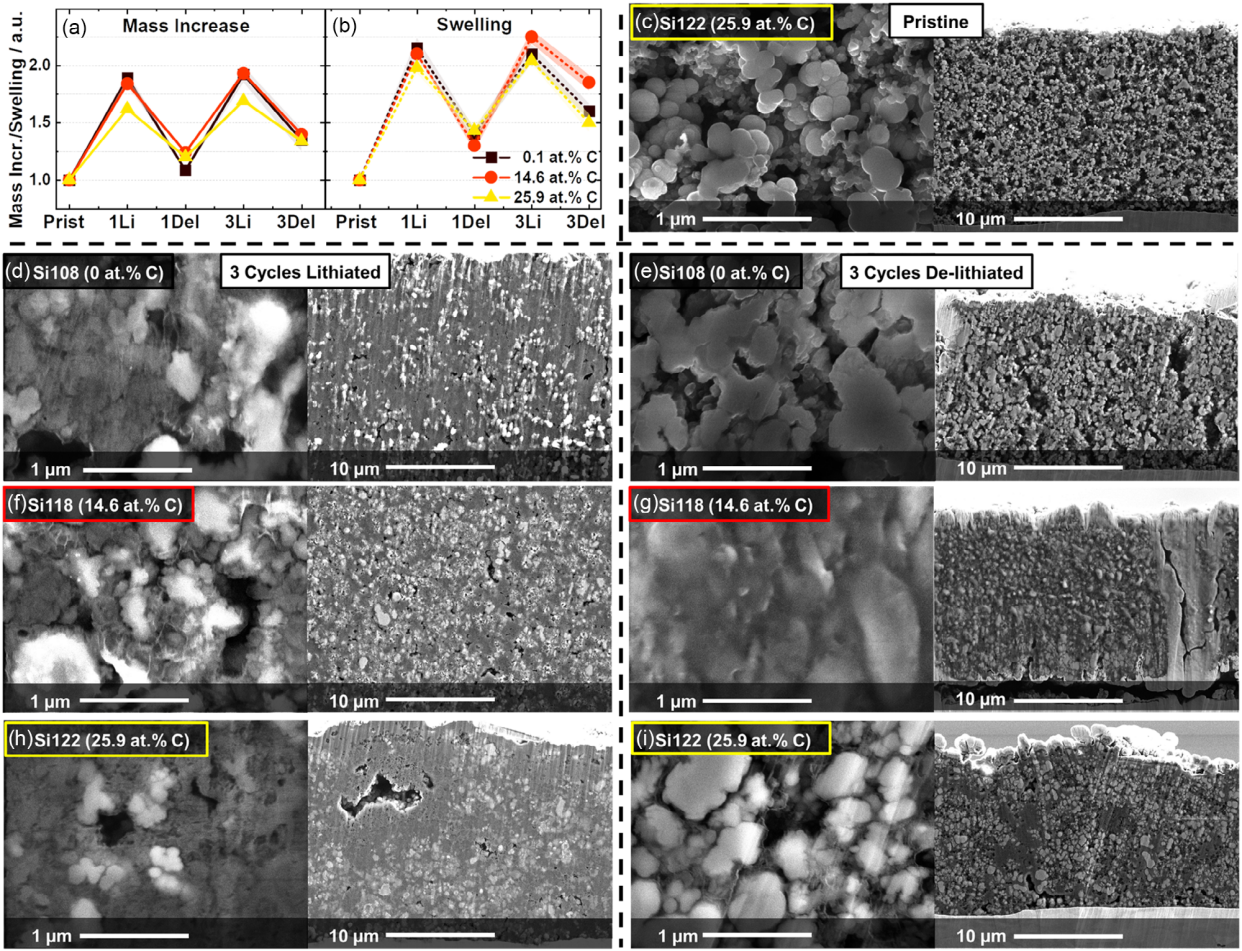
(a,b) Postmortem analysis regarding mass gain and electrode thickness increase measured with a micrometer screw, relative to the electrode before cycling was started. (c–i) Cross‐section views by electron microscope of electrodes, (c) before cycling, and (e,g,i) at delithiation state of third cycle and (d,f,h) lithiation state of third cycle. (d,e) show pure silicon, (f,g) material 118 with 14.6 at.%C, and (h) and (i) material 122 with 25.9 at.% C.

In the next step, the volume change is measured via SEM analysis of electrodes before and after cycling in dependence on the carbon content of the active material. The hypothesis to test is, if volume change is reduced for carbon‐rich materials compared to pure silicon. While generally for lower specific capacities a proportionally lower volume expansion can be expected [8, 56] it remains interesting if a‐SiC_*x*_ with high carbon content will show an initial expansion correlated with the initial lithium consumption. For 3 selected materials, Figure 5a,b show the evaluation of thickness measurements and weighing of the electrodes in different stages of lithiation and cycles, while c–i show SEM micrographs of polished cross‐sections for different materials, pristine and after 3 cycles of lithiation and delithiation.

Regarding the mass gain, the carbon‐rich material (25.9 at.% C) shows an increase lower by 15% compared to pure silicon when lithiated, while when de‐lithiated, the swelling and mass gain are of the same relative magnitude. A similar trend can be observed for evolution of the thickness of the electrode. The maximum swelling is reduced for the carbon‐rich material, while when de‐lithiated again, no clear trend is visible. Apparently, the carbon‐rich materials are swelling between a fully‐ and half‐expanded state. Yet the results are rather weak, due to big deviations between repetition measurements. The morphology and porosity can be estimated from the SEM micrographs, the pristine electrode in Figure 5c is acting as a reference picture to assess differences. Thickness variations cannot directly be compared here, since all electrodes were individual ones with slightly varying thickness from the start. The residual porosity after lithiation in the third cycle is low for all samples, with no obvious differences visible. After delithiation of the third cycle, the pure silicon shows a bigger residual porosity compared to the other materials. A similar trend can be observed regarding particle morphology, while the individual particles can still be well distinguished from pure silicon in de‐lithiated state, for the carbon‐rich material in the de‐lithiated state, the particles still are strongly enlarged and deformed. These results could confirm that a larger part of the lithium is irreversibly bound in the material, keeping it at a rather high level of volume expansion on average. This would be in line with the observations on FCE. Yet the coating layers still seem to be intact, without major defects visible. Material Si118 with 14.63 at.% C, in the middle C‐concentration range, generally shows results in between the two extremes regarding carbon content. Since the residual porosity for the lithiated electrodes is quite low, it is to some extend surprising that the electrodes still perform quite well, even under high charge rates.

### 3.4 In‐Situ Electrochemical Analysis

**FIGURE 6:**
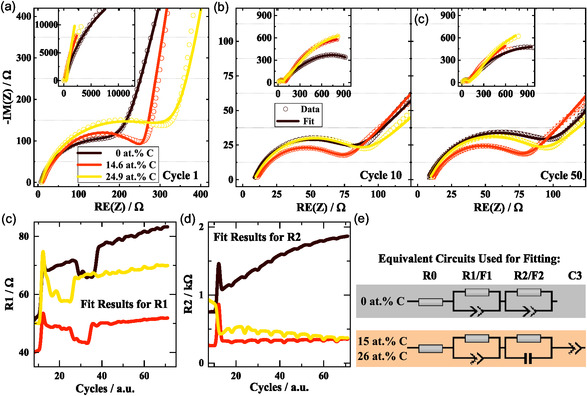
EIS spectra of materials with 0, 14.6, and 24.9 at.% C measured at (a) cycle 1, (b) cycle 10, and (c) cycle 50 in delithiated state at a frequency range from 0.05 Hz to 100 kHz. The circles show the experimental data, while the lines depict the respective fits with an equivalent circuit model. The complete spectra are shown in the insets. (c) Fit results for R1, (d) fit results for R2, and (e) equivalent circuit models used for full evaluation of the fitted parameters are available in Figure SI 9.

Changes on the electrode‐ and particle‐level can also be monitored by EIS, spectra for 3 selected materials at cycle 1, cycle 10, and cycle 50 are presented in Figure 6.

Comparing the 3 materials, the spectra are generally rather similar in structure and measured values. In the first cycle, the pure silicon shows a reduced impedance in the high‐frequency part, but a higher impedance in the low‐frequency part (inset Figure 6a, typically associated with slower diffusion processes in the electrolyte and bulk. For subsequent measurements (cycle 10 and 50), the carbon‐containing materials show a similar or slightly reduced overall impedance compared to the pure silicon, indicating a less severe SEI buildup, and stability of interfaces. Furthermore, the impedance spectra for cycle 10 and 50 are almost identical with only a slight increase in impedance, which shows a good reversibility of the electrode and particle lithiation. This is also reflected in the fitted values of the equivalent‐circuit components (Figure 6e), when plotting them over the cycle number. For the carbon‐rich material, often the parameters are more stable, while they increase the strongest for the pure silicon material. Most notable R1 (often assigned to SEI) and R2 (often assigned to charge transfer processes) show this behavior, as presented in Figure 6c,d. A detailed analysis is available in Figure SI9.

## 4 Conclusion

Obtaining high‐capacity anodes based on silicon for Li‐Ion batteries remains a challenge regarding their lifetime. Diluting the silicon by introducing heteroatoms mixed on an atomic level has been shown to increase overall performance, yet a mechanistic understanding of the processes involved for amorphous, substoichiometric silicon carbide was not available. In this work, a tight mesh of carbon‐containing materials has been produced and analyzed regarding cycling stability and its origins. By direct observations of capacities, efficiencies and potentials, as well as balancing of these effects with the quantity of introduced carbon atoms, following conclusions can be drawn:

The introduction of carbon over‐proportionally reduces the capacity of the material; each carbon atom additionally inertises roughly one silicon atom. The capacity can be completely stabilized over 200 cycles for carbon contents around 25 at.%. Yet one issue is a reduced coulombic efficiency in the first cycles, with each carbon atom also consuming roughly 1 lithium atom during formation, suggesting some kind of additional passive phase forming during the first lithiation. An increased stability can be achieved by stabilizing the interfaces, one important factor being a reduced volume expansion of the material. The synergy of the different effects can effectively stabilize the lithiation and de‐lithiation potentials, and also shows an increased rate‐performance. Postmortem studies indicate that the expansion of the material is happening around the expanded state. While these studies show a superior performance in half‐cell tests, further studies in full cells and blended with graphite may give further insights into the possible applications.

## Supporting Information

Additional supporting information can be found online in the Supporting Information section. **Supporting** **Figure SI1**: Diffractograms of selected samples. **Supporting** **Figure SI2**: Boxplots of the specific resistance measurements of electrodes produced from a‐SiC*_x_* powders with different carbon contents. **Supporting** **Figure SI3**: Cumulative irreversible capacity of the first 3 cycles plotted over the carbon content of the active material. An assumed baseline for a hypothesized SEI formation is drawn in green, while in red a trendline is plotted for an assumed irreversible capacity if each carbon atom of the material would consume 1 lithium atom during formation. **Supporting** **Figure SI4**: Differential capacity plots of the materials with various carbon content from cycle 1 to 260. y‐Axis is adapted for each individual plot. **Supporting** **Figure SI5**: Evaluation of Figure 2 for different carbon contents of the anode material plotted over the cycle number. 200‐260 cycles show a rate test up to 10C. a) average potential difference and b) contribution of the constant current phase to total capacity of cycle. Figure SI6: Schematic of the evaluation of GITT for the determination of the diffusion coefficient. **Supporting** **Figure SI7**: Cyclovoltammetry plots of 3 materials at different scanrates, ranging from 10 to 500 µV/s normalized to the current. **Supporting** **Figure SI8**: Evaluation of Figure SI 7 according to Eq. 2. **Supporting** **Figure SI9**: Evaluation of Figure 6. Equivalent circuit and fitted parameters of each part of the equivalent circuit plotted over cycle number. **Supporting** **Table SI1**: List of all samples used in this study, together with results of powder diffraction, BET analysis and carbon analysis.

## Author Contributions

**Moritz Loewenich**: conceptualization (lead), investigation (lead), methodology (lead), visualization (lead), writing – original draft (lead), writing – review & editing (equal). **Hartmut Wiggers**: funding acquisition (lead), project administration (lead), supervision (lead), writing – review & editing (equal).

## Funding

This work was supported by the German Ministry of Economy and Climate Action [grant number 03EI3027A].

## Conflicts of Interest

The authors declare no conflicts of interest.

## Supporting information

Supplementary Material

## Data Availability

Data are available on reasonable request.
