# Supplementary material for: Silicon‐Rich Amorphous SiC x for the Lithium‐Ion Batteries: How Does Strong Carbon Doping Affect the Lithiation Behavior and Electrochemical Performance?
Source: ChemSusChem. 2026 Apr 5;19(7):e202502723. doi: 10.1002/cssc.202502723 (PMC13050542; doi:10.1002/cssc.202502723)
Supplement: Supplementary file 1 — Supplementary Material [file CSSC-19-e202502723-s001.pdf]

Supplementary Information

Silicon rich amorphous SiC_x for the lithium-ion batteries: How does strong carbon doping affect the lithiation behavior and electrochemical performance?

Moritz Loewenich[1], Hartmut Wiggers[1,2,z]

[1] EMPI, Institute for Energy and Materials Processes – Reactive Fluids, University of Duisburg-Essen, Duisburg, Germany

[2] CENIDE, Center for Nanointegration Duisburg-Essen, University of Duisburg-Essen, Duisburg, Germany

[z] Corresponding Author E-mail Address: hartmut.wiggers@uni-due.de

Table SI 1: List of all samples used in this study, together with results of powder diffraction, BET analysis and carbon analysis.

**Material ID**	**C (D1a) / %**	**Amorphous Fraction / vol %**	**BET Surface / m^2/g**	**BET Equivalent Diameter / nm**	**C- Cont. / wt%**	**Error / wt. %**	**C-Cont. / At. %**
**108**	0.0	95	9.60	268	0.07	0.01	0.16
**109**	0.1				0.38	0.01	0.89
**110**	0.3		10.5	246	0.61	0.01	1.41
**111**	0.6				1.24	0.02	2.85
**112**	0.9	100	10.4	248	1.76	0.02	4.01
**113**	1.2				2.38	0.02	5.38
**114**	1.6		11.7	219	3.1	0.06	6.95
**115**	2.2				4.05	0.03	8.97
**116**	2.7	100	12.5	205	4.81	0.06	10.6
**117**	3.3				5.59	0.02	12.1
**118**	4.1		13.3	193	6.84	0.21	14.6
**119**	5.0				7.83	0.16	16.5
**120**	6.2	100	14.4	178	9.21	0.17	19.1
**121**	7.7				11.3	0.29	22.9
**122**	9.4		13.1	197	13	0.18	25.9
**123**	11.2				14.8	0.06	28.8
**124**	13.3	100	12.1	213	16.9	0.22	32.2
**125**	16.8				20.4	0.03	37.4

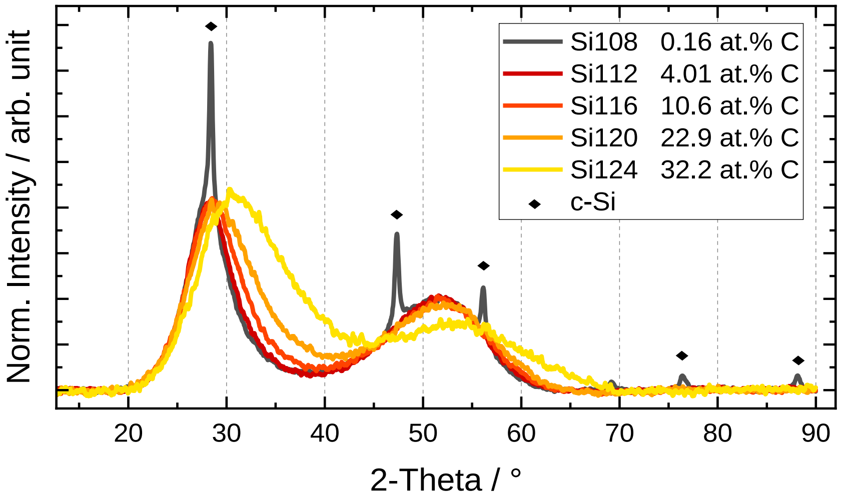

Figure SI 1: Diffractograms of selected samples

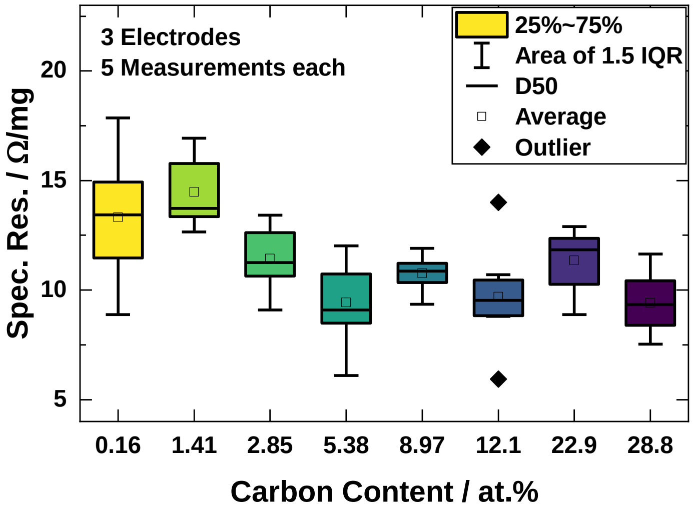

Figure SI 2: Boxplots of the specific resistance measurements of electrodes produced from a-SiC_x powders with different carbon contents.

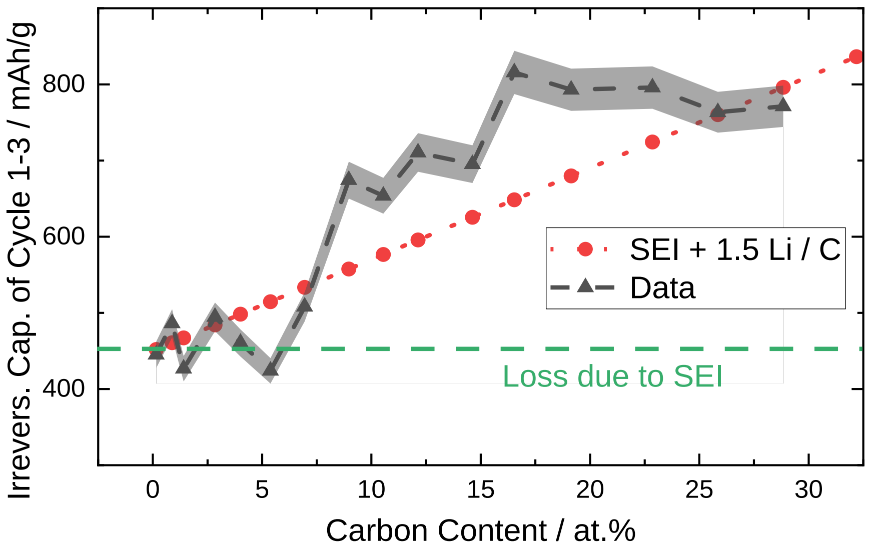

Figure SI 3: Cumulative irreversible capacity of the first 3 cycles plotted over the carbon content of the active material. An assumed baseline for a hypothesized SEI formation is drawn in green, while in red a trendline is plotted for an assumed irreversible capacity if each carbon atom of the material would consume 1 lithium atom during formation.

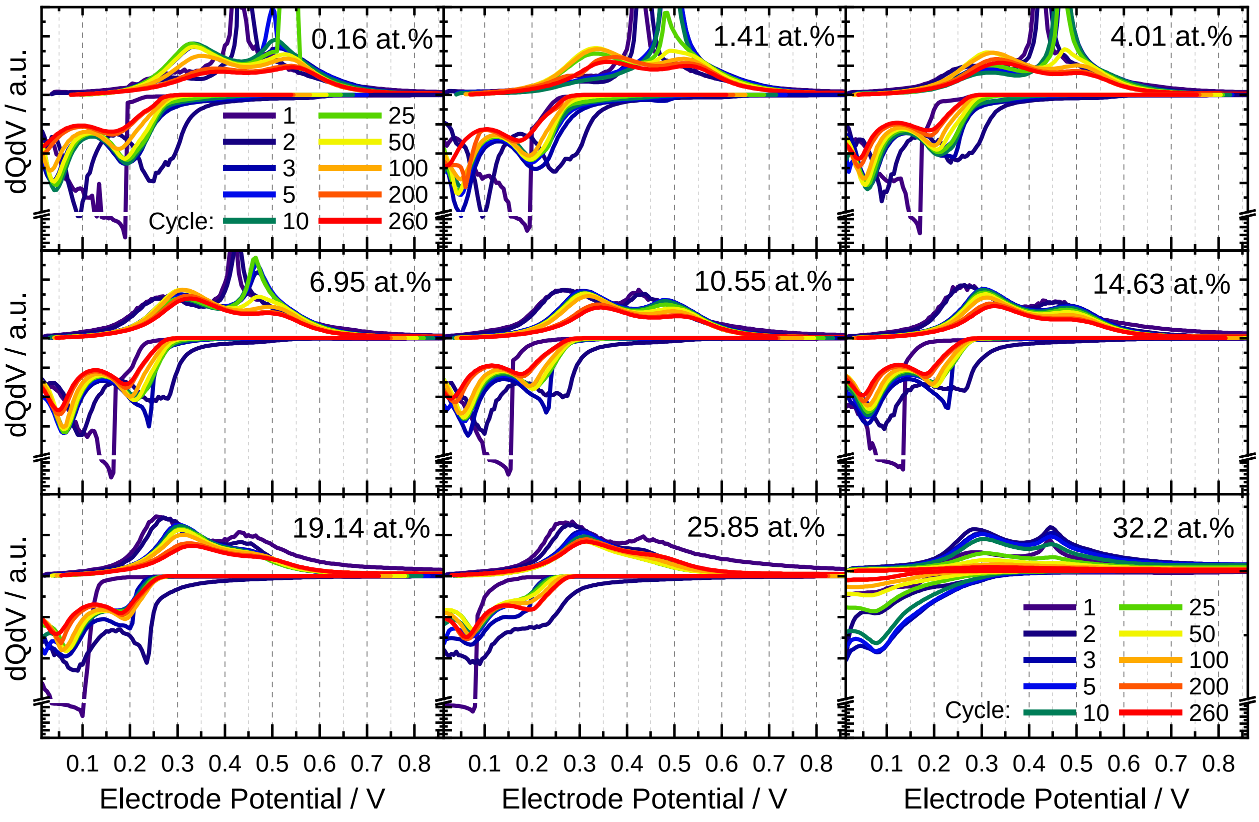

Figure SI 4: Differential capacity plots of the materials with various carbon content from cycle 1 to 260. y-Axis is adapted for each individual plot.

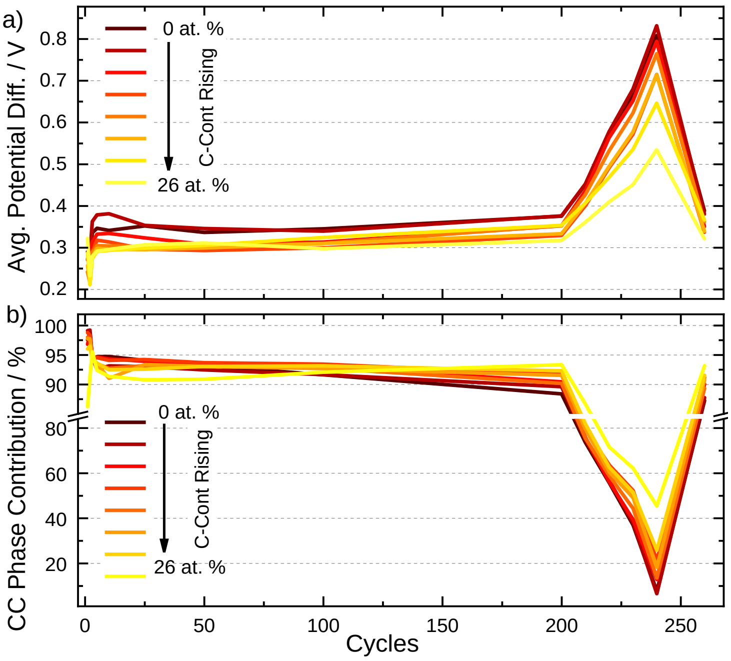

Figure SI 5: Evaluation of Figure 2 for different carbon contents of the anode material plotted over the cycle number. 200-260 cycles show a rate test up to 10C. a) average potential difference and b) contribution of the constant current phase to total capacity of cycle.

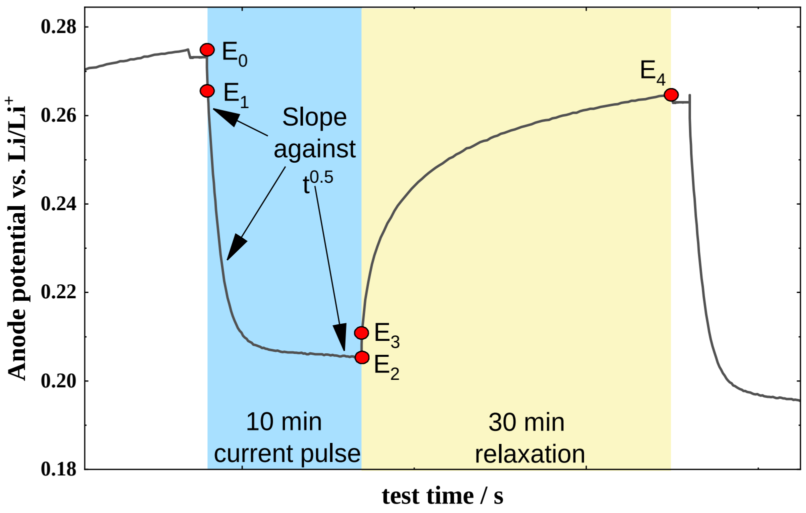

Figure SI 6: Schematic of the evaluation of GITT for the determination of the diffusion coefficient.

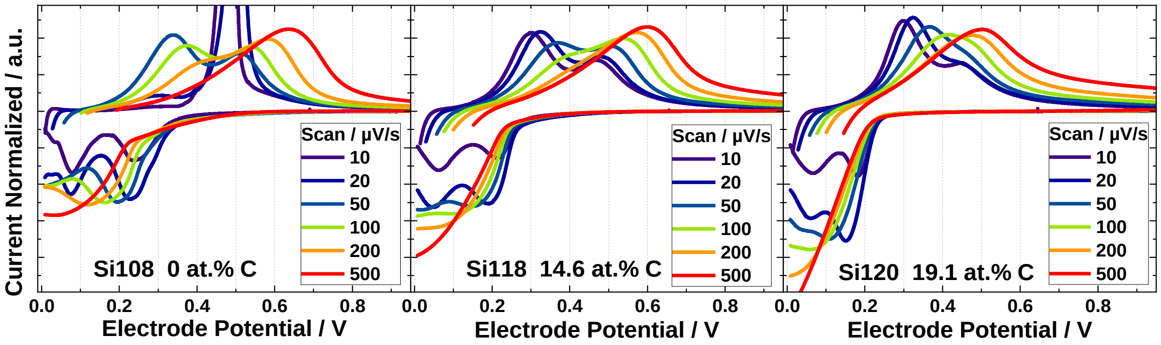

Figure SI 7: Cyclovoltammetry plots of 3 materials at different scanrates, ranging from 10 to 500 µV/s normalized to the current.

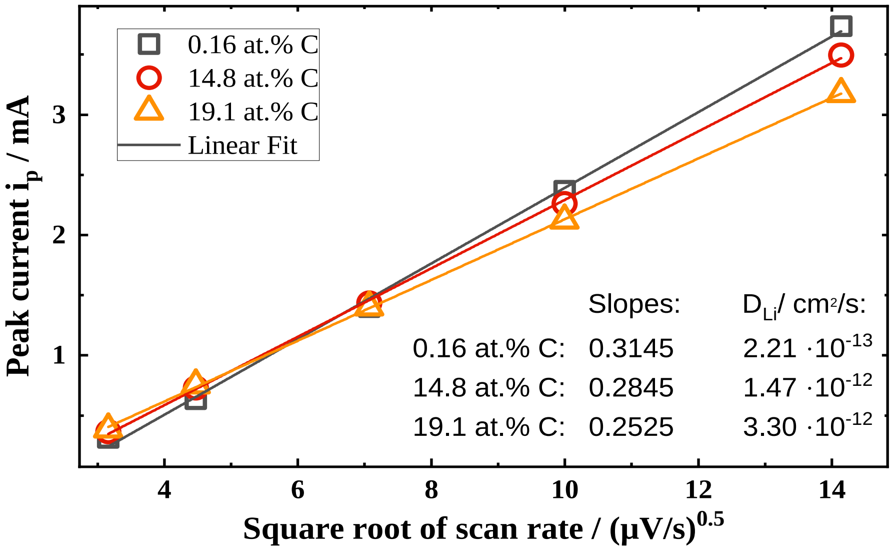

Figure SI 8: Evaluation of Figure SI 7 according to Eq. 2.

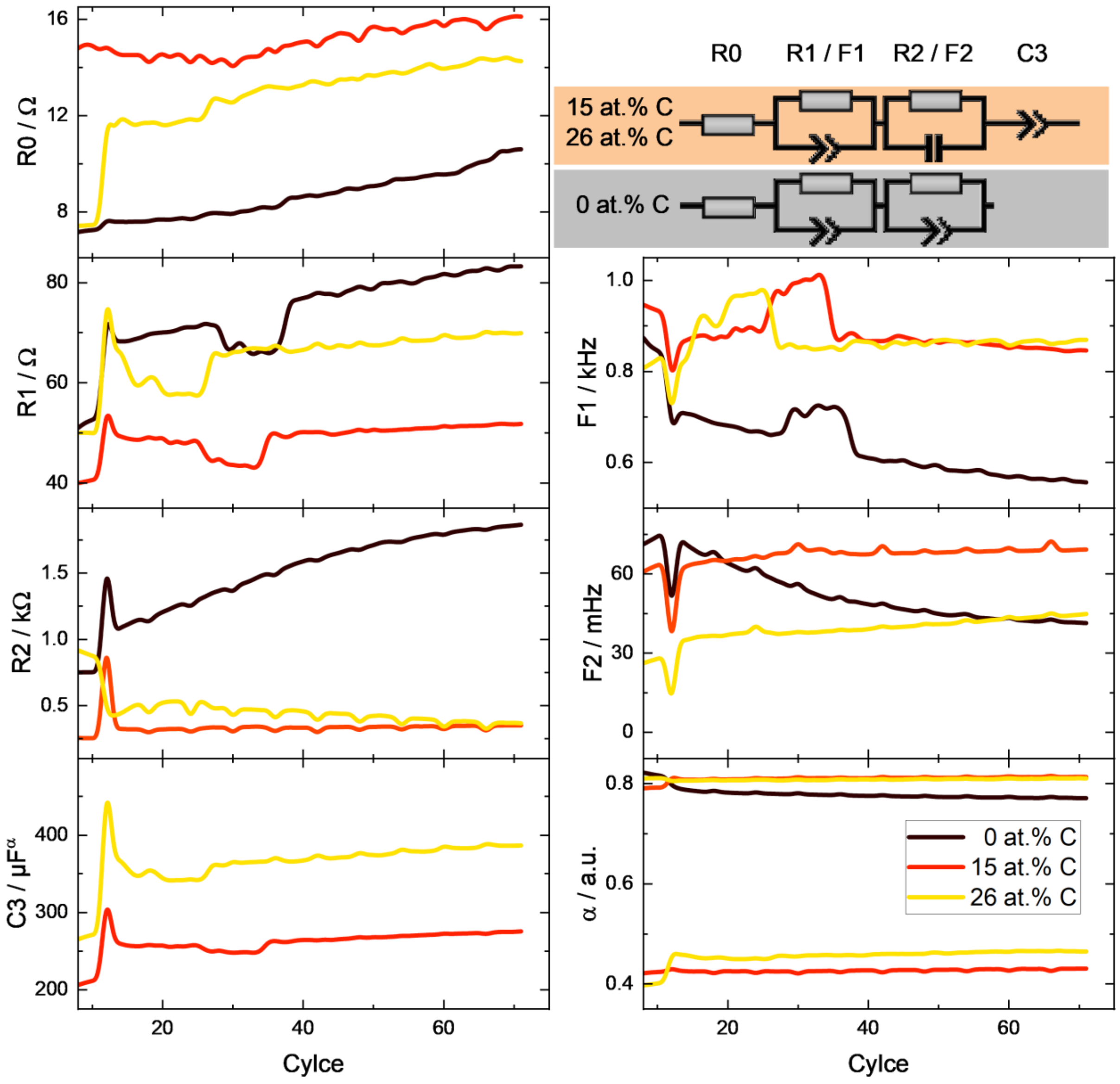

Figure SI 9: Evaluation of Figure 6. Equivalent circuit and fitted parameters of each part of the equivalent circuit plotted over cycle number.
